# Supplementary material for: Phylogenetic Analysis of Some Species of the Anopheles hyrcanus Group (Diptera: Culicidae) in China Based on Complete Mitochondrial Genomes
Source: Genes (Basel). 2023 Jul 16;14(7):1453. doi: 10.3390/genes14071453 (PMC10379722; doi:10.3390/genes14071453)
Supplement: Supplementary file 1 [file genes-14-01453-s001.zip › Fig. S1.pdf]

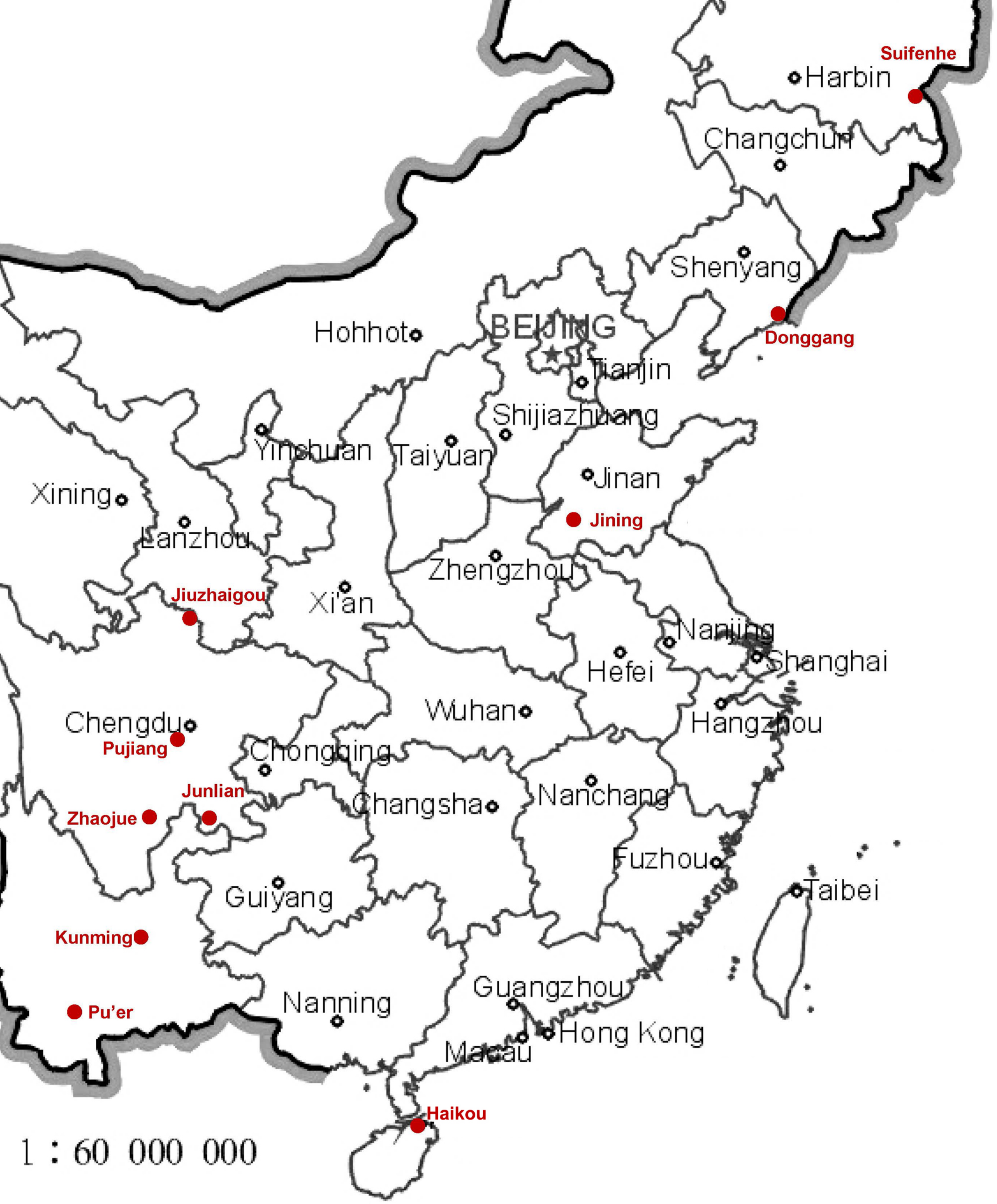
Suifenhe
Harbin
Changchun
Shenyang
Donggang
BEIJING
Hohhot
Tianjin
Shijiazhuang
Yinchuan
Taiyuan
Jinan
Xining
Lanzhou
Jining
Zhengzhou
Jiuzhaigou
Xi'an
Nanjing
Shanghai
Hefei
Wuhan
Hangzhou
Chengdu
Pujiang
Chongqing
Junlian
Zhaojue
Changsha
Nanchang
Fuzhou
Guiyang
Taibei
Kunming
Guangzhou
Nanning
Pu'er
Hong Kong
Macau
Haikou
1 : 60 000 000
