# Supplementary material for: Phylogenetic Analysis of Some Species of the Anopheles hyrcanus Group (Diptera: Culicidae) in China Based on Complete Mitochondrial Genomes
Source: Genes (Basel). 2023 Jul 16;14(7):1453. doi: 10.3390/genes14071453 (PMC10379722; doi:10.3390/genes14071453)
Supplement: Supplementary file 1 [file genes-14-01453-s001.zip › Fig. S2.pdf]

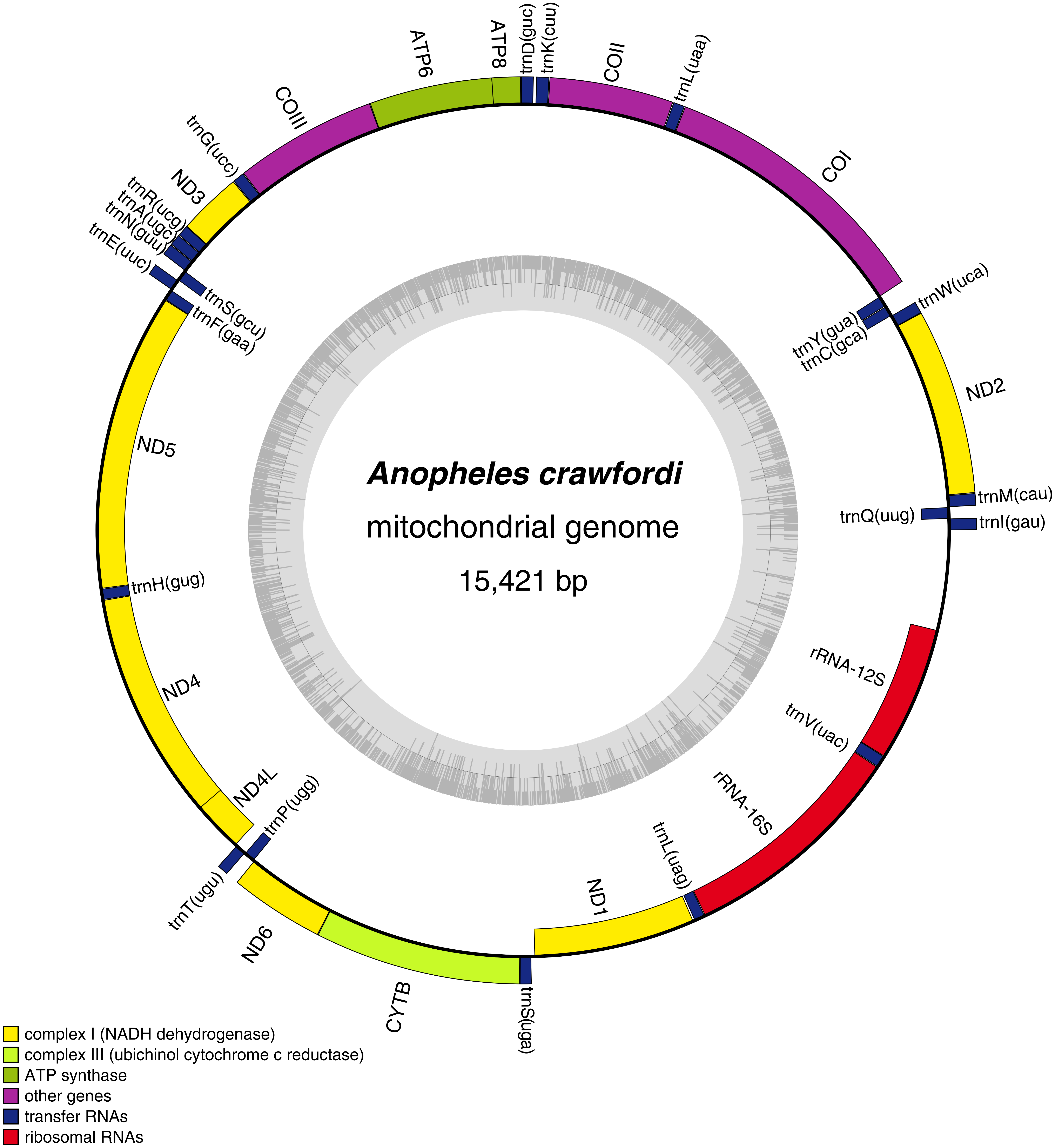
Anopheles crawfordi
mitochondrial genome
15,421 bp
ATP6
ATP8
trnD(guc)
trnK(cuu)
COII
trnL(uaa)
COI
COIII
trnG(ucc)
ND3
trnR(ucg)
trnA(ugc)
trnN(guu)
trnE(uuc)
trnS(gcu)
trnF(gaa)
trnW(uca)
trnY(gua)
trnC(gca)
ND2
ND5
trnM(cau)
trnQ(uug)
trnI(gau)
trnH(gug)
rRNA-12S
ND4
trnV(uac)
ND4L
trnP(ugg)
rRNA-16S
trnL(uag)
ND1
trnT(ugu)
ND6
CYTB
trnS(uga)
complex I (NADH dehydrogenase)
complex III (ubichinol cytochrome c reductase)
ATP synthase
other genes
transfer RNAs
ribosomal RNAs

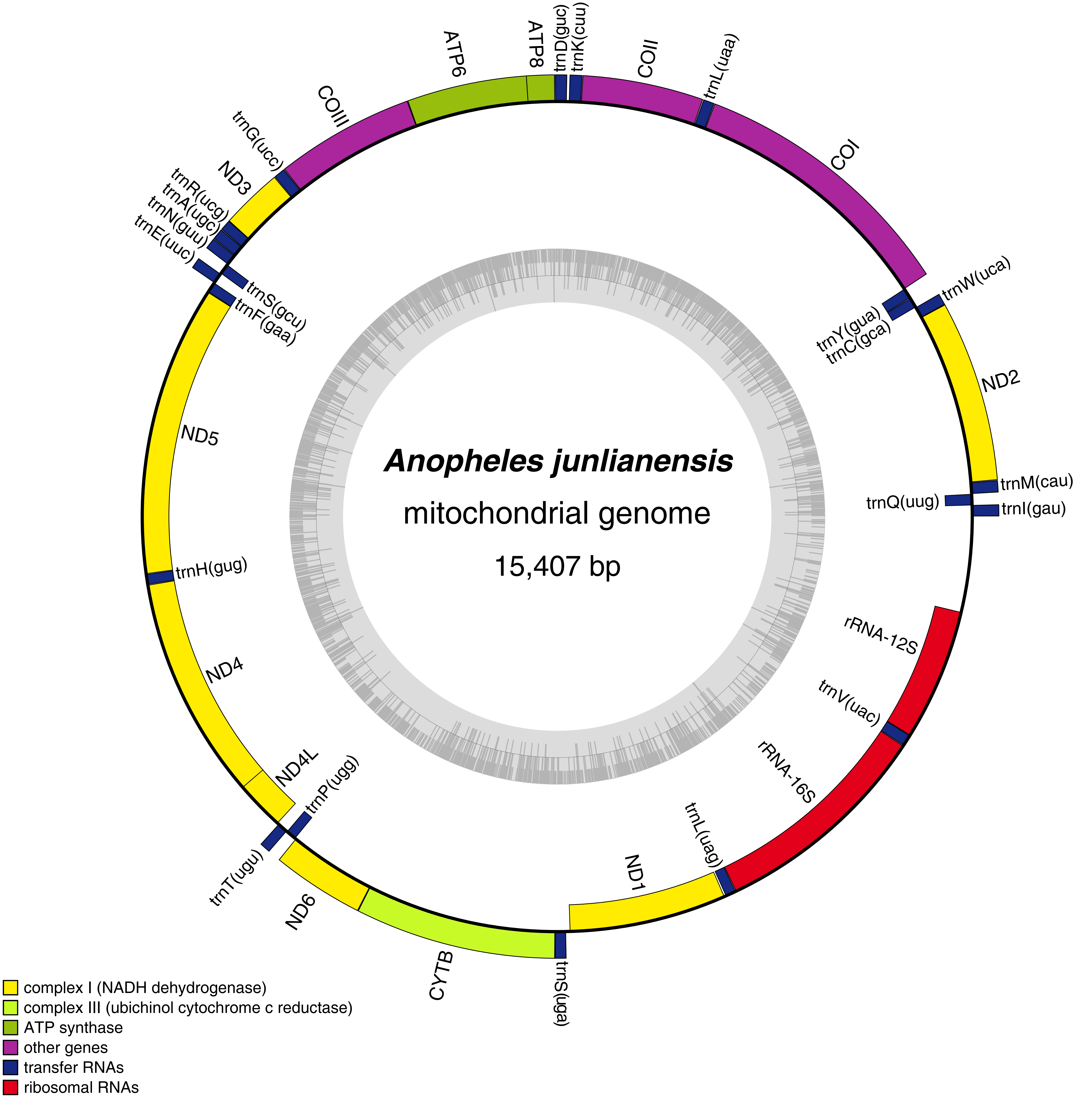
Anopheles junlianensis
mitochondrial genome
15,407 bp
COI
COII
trnL(uaa)
trnK(cuu)
trnD(guc)
ATP8
ATP6
COIII
trnG(ucc)
ND3
trnR(ucg)
trnA(ugc)
trnN(guu)
trnE(uuc)
trnS(gcu)
trnF(gaa)
ND5
trnH(gug)
ND4
ND4L
trnP(ugg)
trnT(ugu)
ND6
CYTB
trnS(uga)
ND1
trnL(uag)
rRNA-16S
trnV(uac)
rRNA-12S
trnI(gau)
trnQ(uug)
trnM(cau)
ND2
trnW(uca)
trnC(gca)
trnY(gua)
complex I (NADH dehydrogenase)
complex III (ubichinol cytochrome c reductase)
ATP synthase
other genes
transfer RNAs
ribosomal RNAs

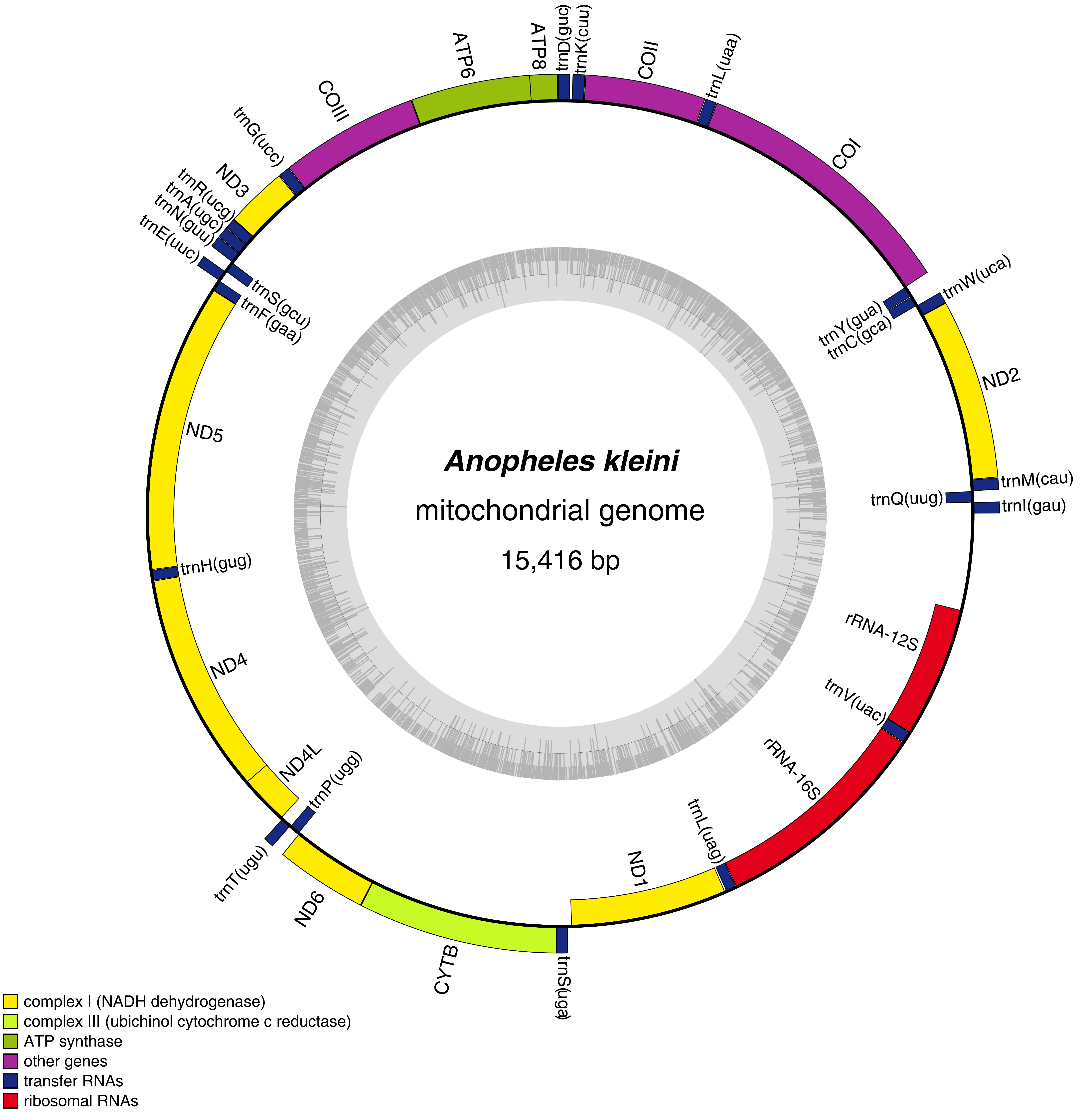
Anopheles kleini
mitochondrial genome
15,416 bp
COI
trnL(uaa)
COII
trnK(cuu)
trnD(guc)
ATP8
ATP6
COIII
trnG(ucc)
ND3
trnR(ucg)
trnA(ugc)
trnN(guu)
trnE(uuc)
trnS(gcu)
trnF(gaa)
ND5
trnH(gug)
ND4
ND4L
trnP(ugg)
trnT(ugu)
ND6
CYTB
trnS(uga)
ND1
trnL(uag)
rRNA-16S
trnV(uac)
rRNA-12S
trnI(gau)
trnQ(uug)
trnM(cau)
ND2
trnW(uca)
trnY(gua)
trnC(gca)
complex I (NADH dehydrogenase)
complex III (ubichinol cytochrome c reductase)
ATP synthase
other genes
transfer RNAs
ribosomal RNAs

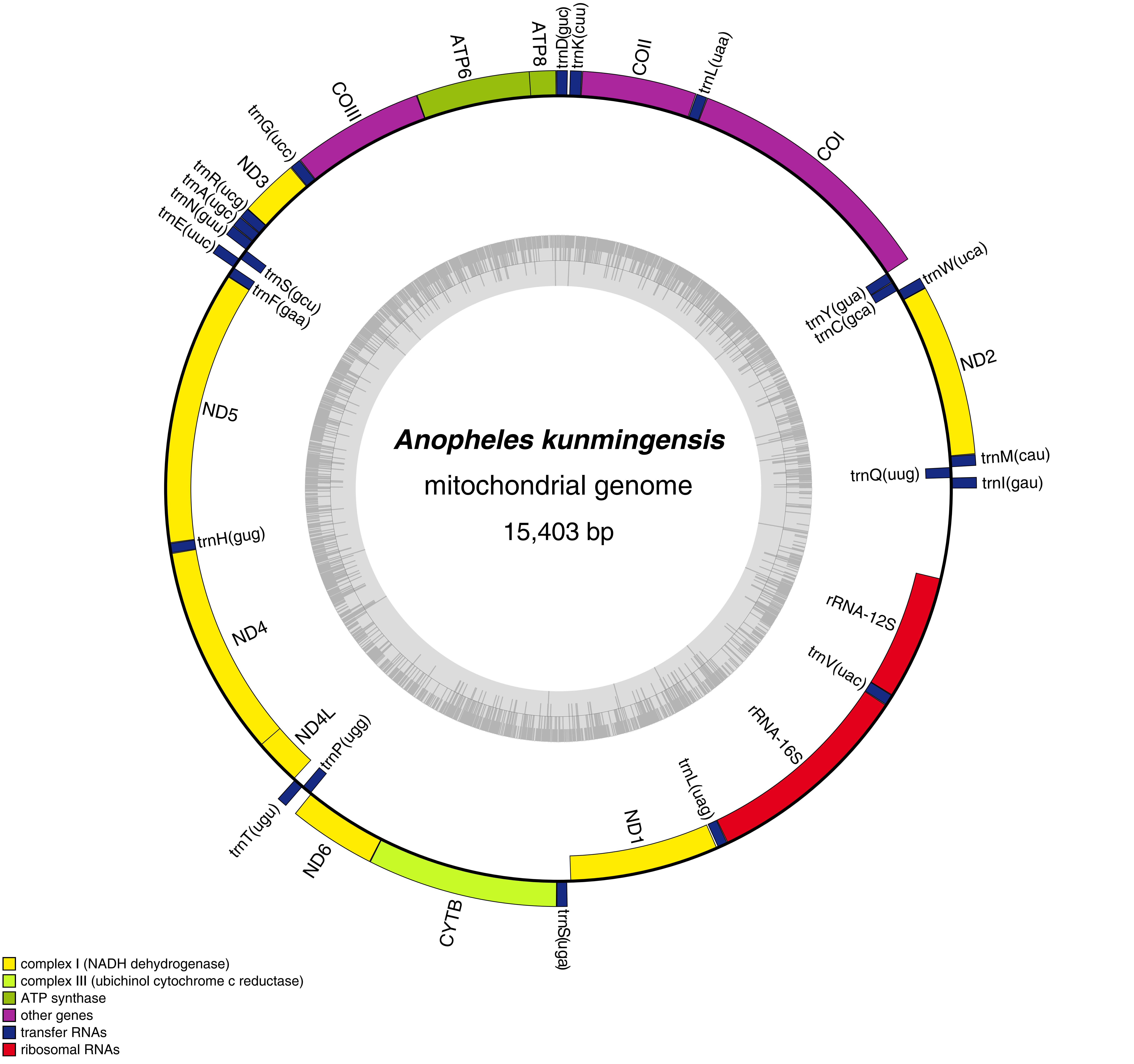

Anopheles kunmingensis
mitochondrial genome
15,403 bp
COI
COII
trnL(uaa)
trnK(cuu)
trnD(guc)
ATP8
ATP6
COIII
trnG(ucc)
ND3
trnR(ucg)
trnA(ugc)
trnN(guu)
trnE(uuc)
trnS(gcu)
trnF(gaa)
ND5
trnH(gug)
ND4
ND4L
trnP(ugg)
trnT(ugu)
ND6
CYTB
trnS(uga)
ND1
trnL(uag)
rRNA-16S
trnV(uac)
rRNA-12S
trnI(gau)
trnQ(uug)
trnM(cau)
ND2
trnW(uca)
trnY(gua)
trnC(gca)
complex I (NADH dehydrogenase)
complex III (ubichinol cytochrome c reductase)
ATP synthase
other genes
transfer RNAs
ribosomal RNAs

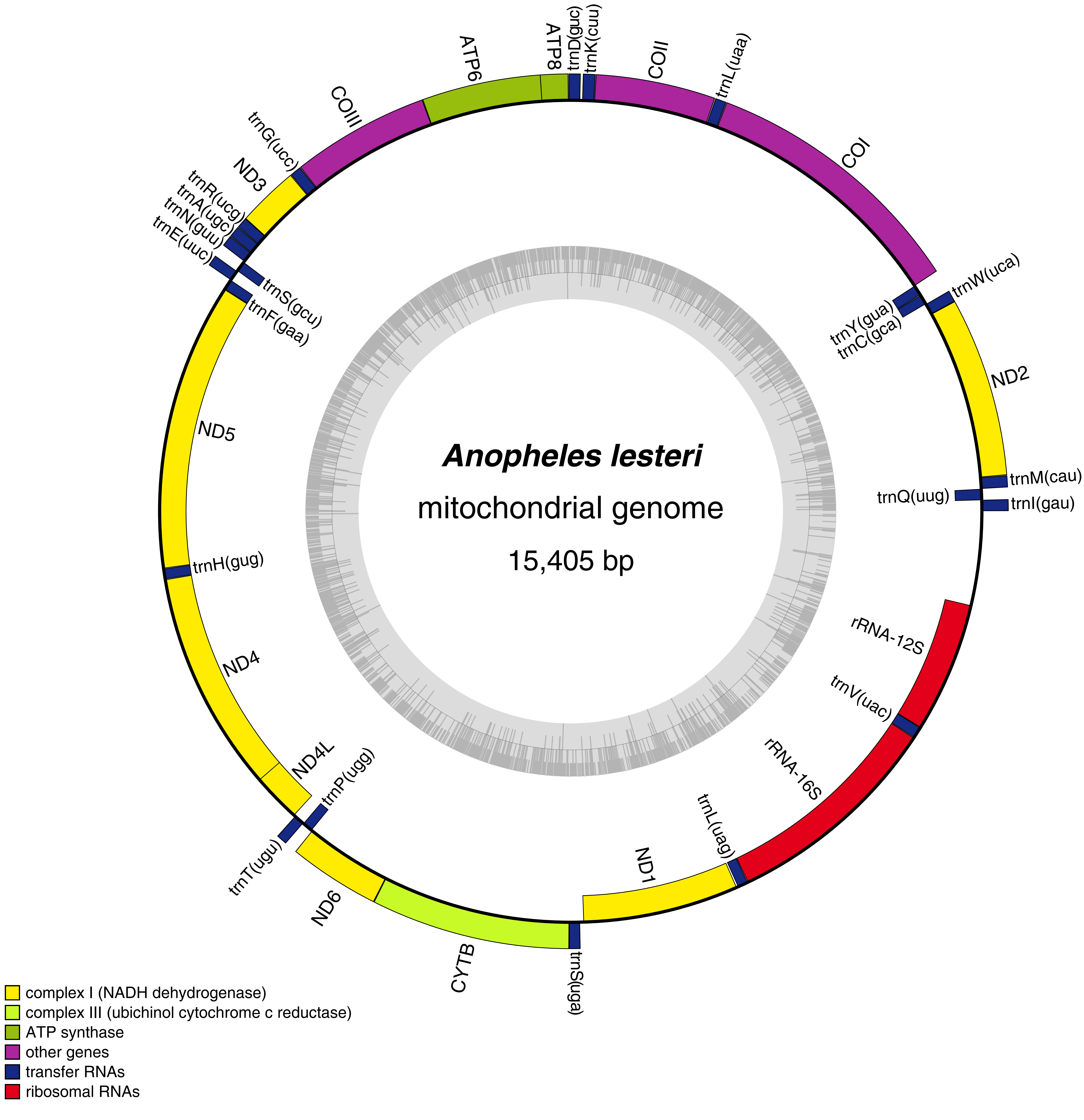
Anopheles lesteri
mitochondrial genome
15,405 bp
ATP6
ATP8
trnD(guc)
trnK(cuu)
COII
trnL(uaa)
COI
COIII
trnG(ucc)
ND3
trnR(ucg)
trnA(ugc)
trnN(guu)
trnE(uuc)
trnS(gcu)
trnF(gaa)
trnW(uca)
trnY(gua)
trnC(gca)
ND2
ND5
trnM(cau)
trnQ(uug)
trnI(gau)
trnH(gug)
rRNA-12S
ND4
trnV(uac)
rRNA-16S
ND4L
trnP(ugg)
trnL(uag)
ND1
trnT(ugu)
ND6
CYTB
trnS(uga)
complex I (NADH dehydrogenase)
complex III (ubichinol cytochrome c reductase)
ATP synthase
other genes
transfer RNAs
ribosomal RNAs

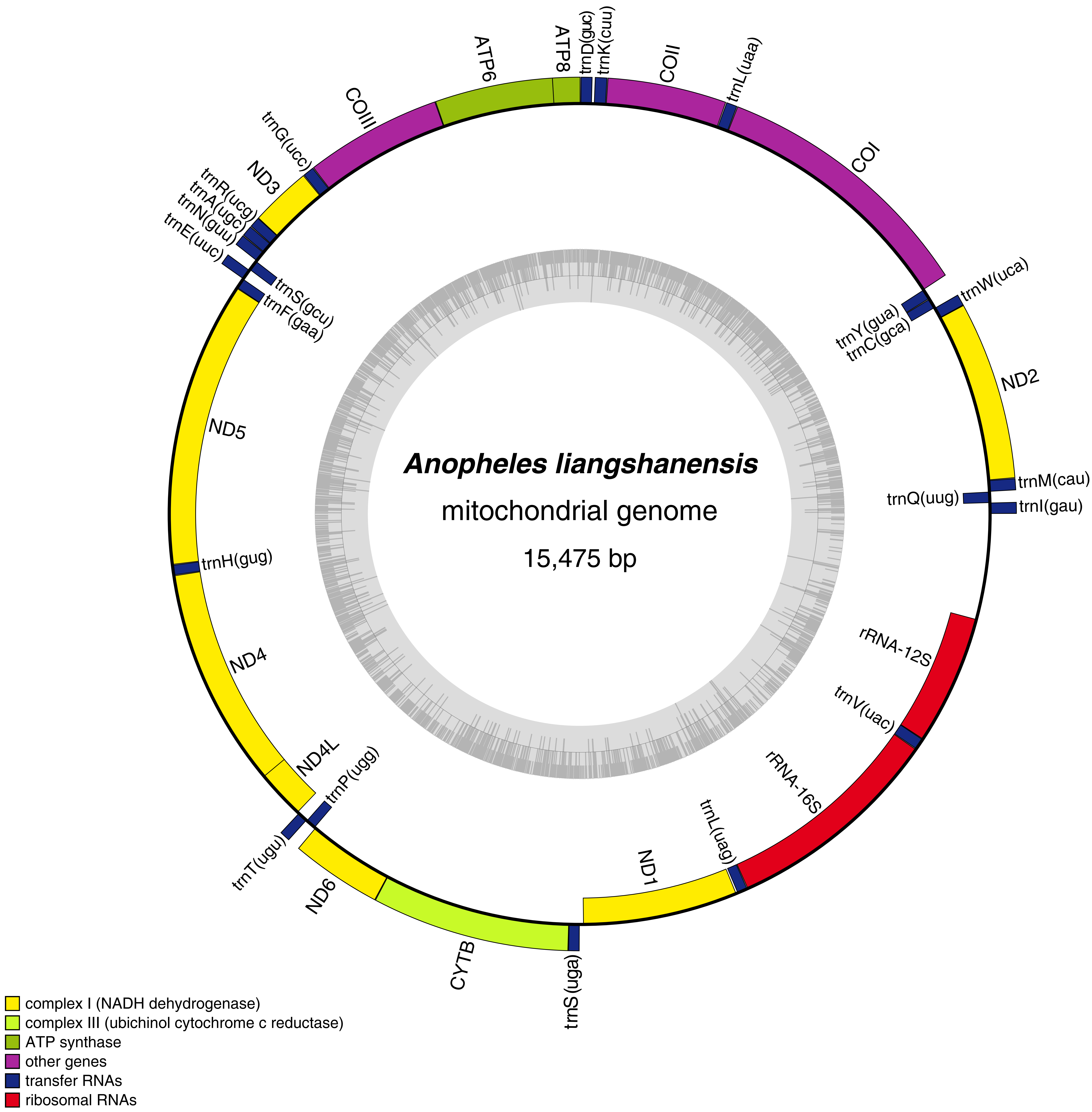

Anopheles liangshanensis
mitochondrial genome
15,475 bp
trnD(guc)
trnK(cuu)
ATP8
ATP6
COII
trnL(uaa)
COIII
trnG(ucc)
COI
ND3
trnR(ucg)
trnA(ugc)
trnN(guu)
trnE(uuc)
trnS(gcu)
trnF(gaa)
trnW(uca)
trnY(gua)
trnC(gca)
ND2
ND5
trnM(cau)
trnQ(uug)
trnI(gau)
trnH(gug)
rRNA-12S
ND4
trnV(uac)
ND4L
trnP(ugg)
rRNA-16S
trnL(uag)
trnT(ugu)
ND1
ND6
CYTB
trnS(uga)
complex I (NADH dehydrogenase)
complex III (ubichinol cytochrome c reductase)
ATP synthase
other genes
transfer RNAs
ribosomal RNAs

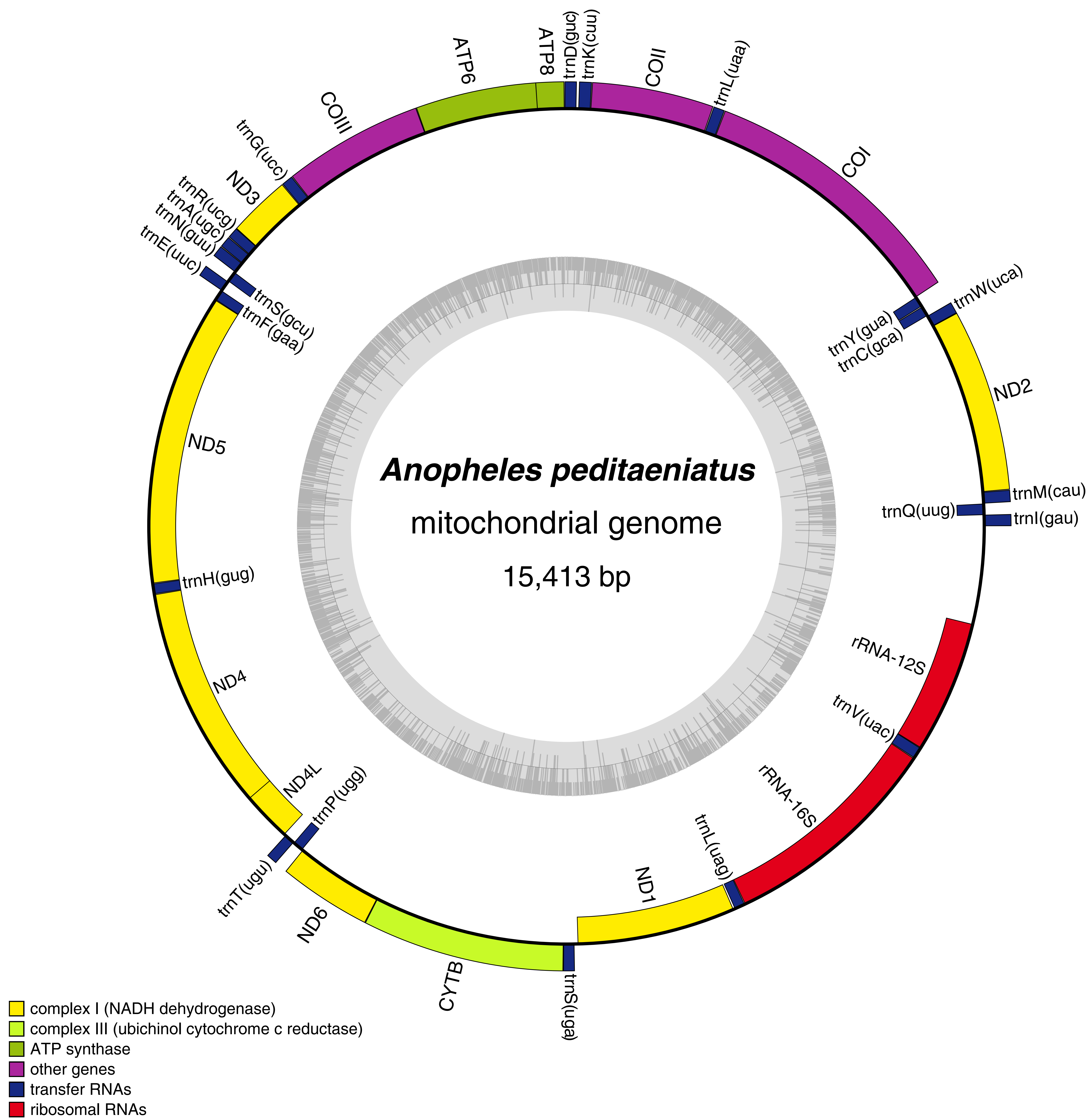

Anopheles peditaeniatus
mitochondrial genome
15,413 bp
trnD(guc)
trnK(cuu)
ATP8
ATP6
COII
trnL(uaa)
COIII
trnG(ucc)
COI
ND3
trnR(ucg)
trnA(ugc)
trnN(guu)
trnE(uuc)
trnS(gcu)
trnF(gaa)
trnW(uca)
trnY(gua)
trnC(gca)
ND2
ND5
trnM(cau)
trnQ(uug)
trnI(gau)
trnH(gug)
rRNA-12S
ND4
trnV(uac)
ND4L
trnP(ugg)
rRNA-16S
trnL(uag)
ND1
trnT(ugu)
ND6
CYTB
trnS(uga)
complex I (NADH dehydrogenase)
complex III (ubichinol cytochrome c reductase)
ATP synthase
other genes
transfer RNAs
ribosomal RNAs

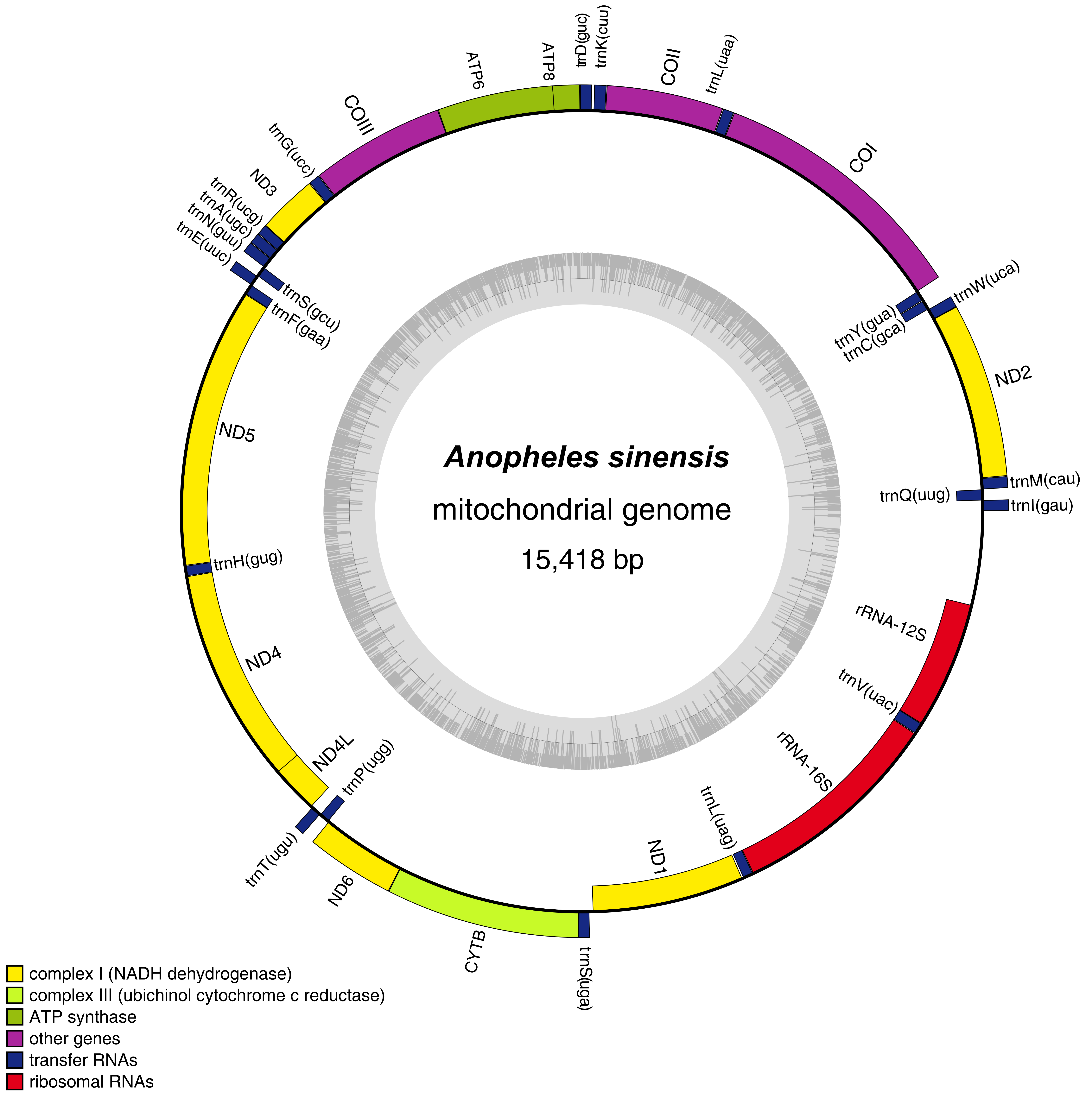
Anopheles sinensis
mitochondrial genome
15,418 bp
COI
COII
COIII
ATP6
ATP8
trnD(guc)
trnK(cuu)
trnL(uaa)
trnG(ucc)
ND3
trnR(ucg)
trnA(ugc)
trnN(guu)
trnE(uuc)
trnS(gcu)
trnF(gaa)
ND5
trnH(gug)
ND4
ND4L
trnP(ugg)
trnT(ugu)
ND6
CYTB
trnS(uga)
ND1
trnL(uag)
rRNA-16S
trnV(uac)
rRNA-12S
trnI(gau)
trnQ(uug)
trnM(cau)
ND2
trnW(uca)
trnY(gua)
trnC(gca)
complex I (NADH dehydrogenase)
complex III (ubichinol cytochrome c reductase)
ATP synthase
other genes
transfer RNAs
ribosomal RNAs

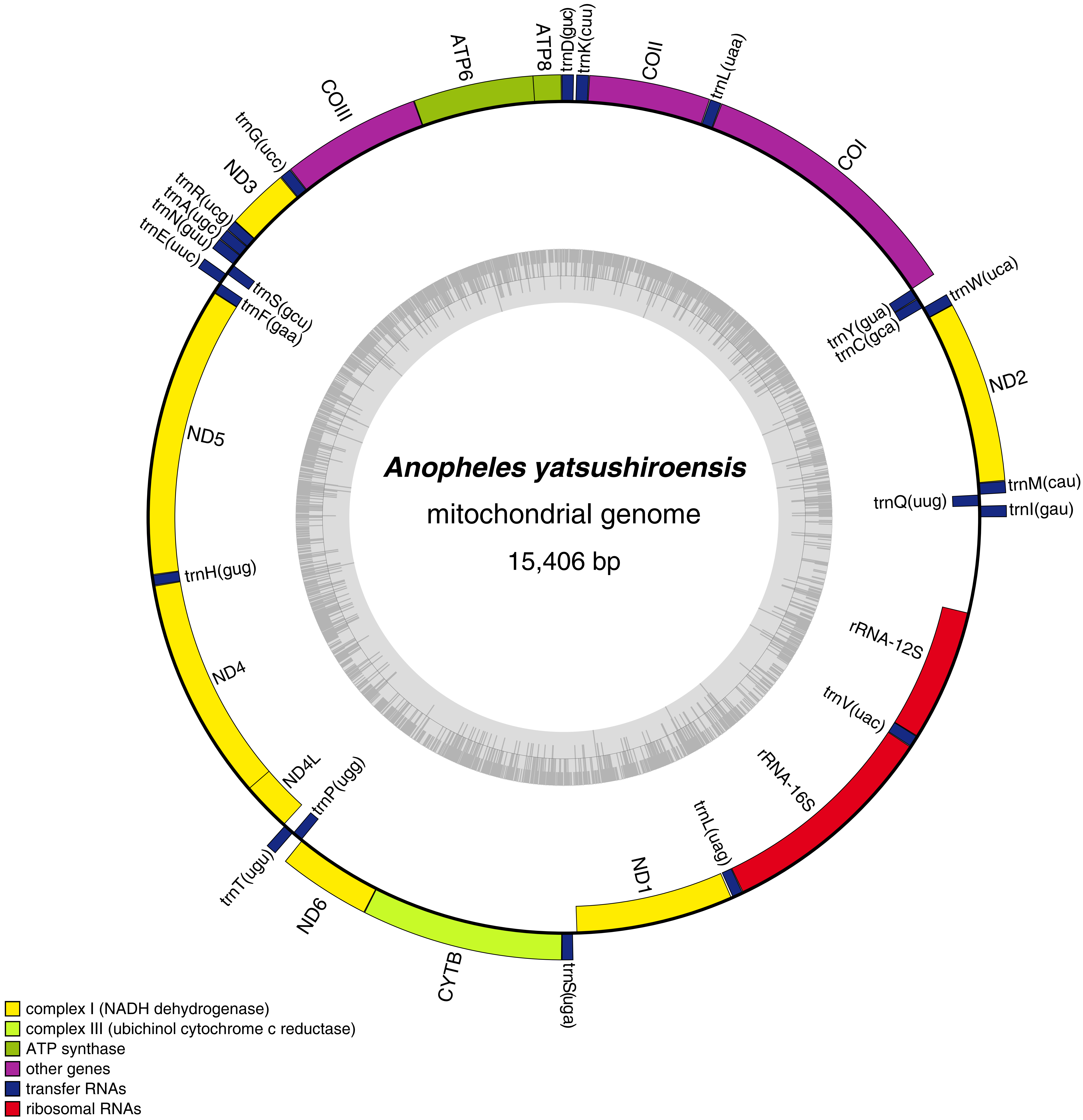
Anopheles yatsushiroensis
mitochondrial genome
15,406 bp
trnD(guc)
trnK(cuu)
COII
trnL(uaa)
COI
trnW(uca)
trnY(gua)
trnC(gca)
ND2
trnM(cau)
trnQ(uug)
trnI(gau)
rRNA-12S
trnV(uac)
rRNA-16S
trnL(uag)
ND1
trnS(uga)
CYTB
ND6
trnT(ugu)
trnP(ugg)
ND4L
ND4
trnH(gug)
ND5
trnF(gaa)
trnS(gcu)
trnE(uuc)
trnN(guu)
trnA(ugc)
trnR(ucg)
ND3
trnG(ucc)
COIII
ATP6
ATP8
complex I (NADH dehydrogenase)
complex III (ubichinol cytochrome c reductase)
ATP synthase
other genes
transfer RNAs
ribosomal RNAs
