# Supplementary material for: Phylogenetic Analysis of Some Species of the Anopheles hyrcanus Group (Diptera: Culicidae) in China Based on Complete Mitochondrial Genomes
Source: Genes (Basel). 2023 Jul 16;14(7):1453. doi: 10.3390/genes14071453 (PMC10379722; doi:10.3390/genes14071453)

An. belenrae

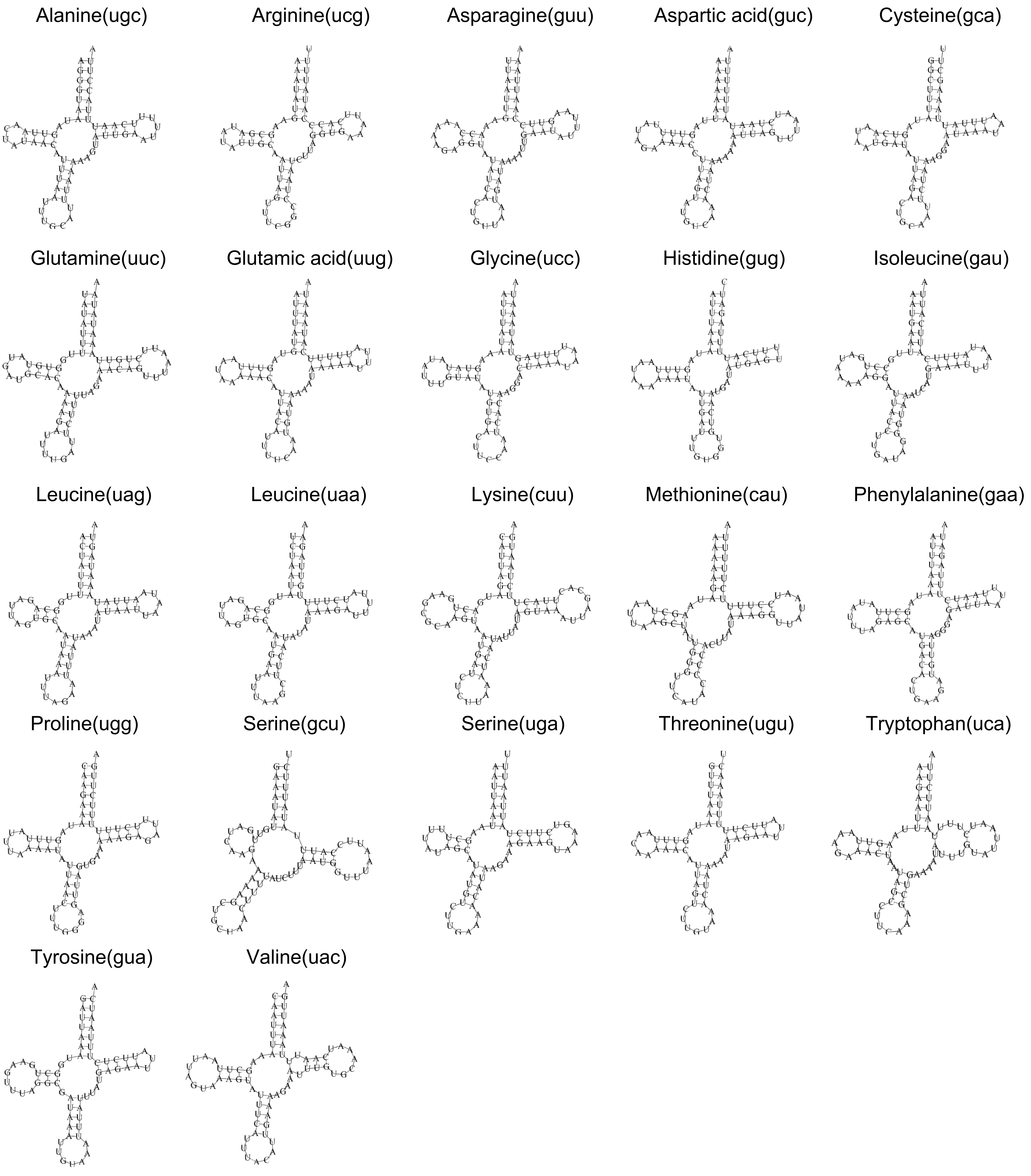
Alanine(ugc)
Arginine(ucg)
Asparagine(guu)
Aspartic acid(guc)
Cysteine(gca)
Glutamine(uuc)
Glutamic acid(uug)
Glycine(ucc)
Histidine(gug)
Isoleucine(gau)
Leucine(uag)
Leucine(uaa)
Lysine(cuu)
Methionine(cau)
Phenylalanine(gaa)
Proline(ugg)
Serine(gcu)
Serine(uga)
Threonine(ugu)
Tryptophan(uca)
Tyrosine(gua)
Valine(uac)

An. crawfordi

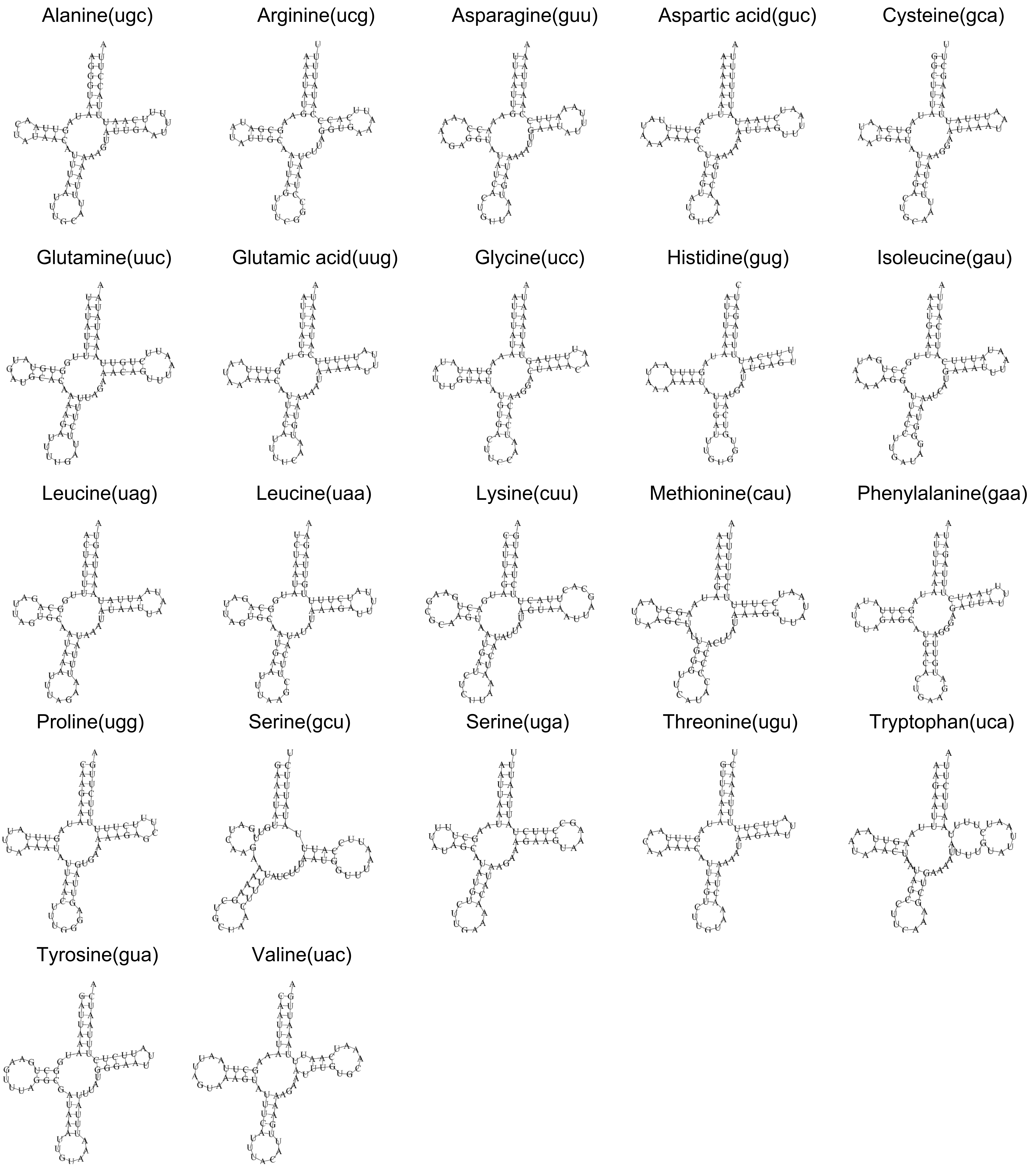

An. junlianensis

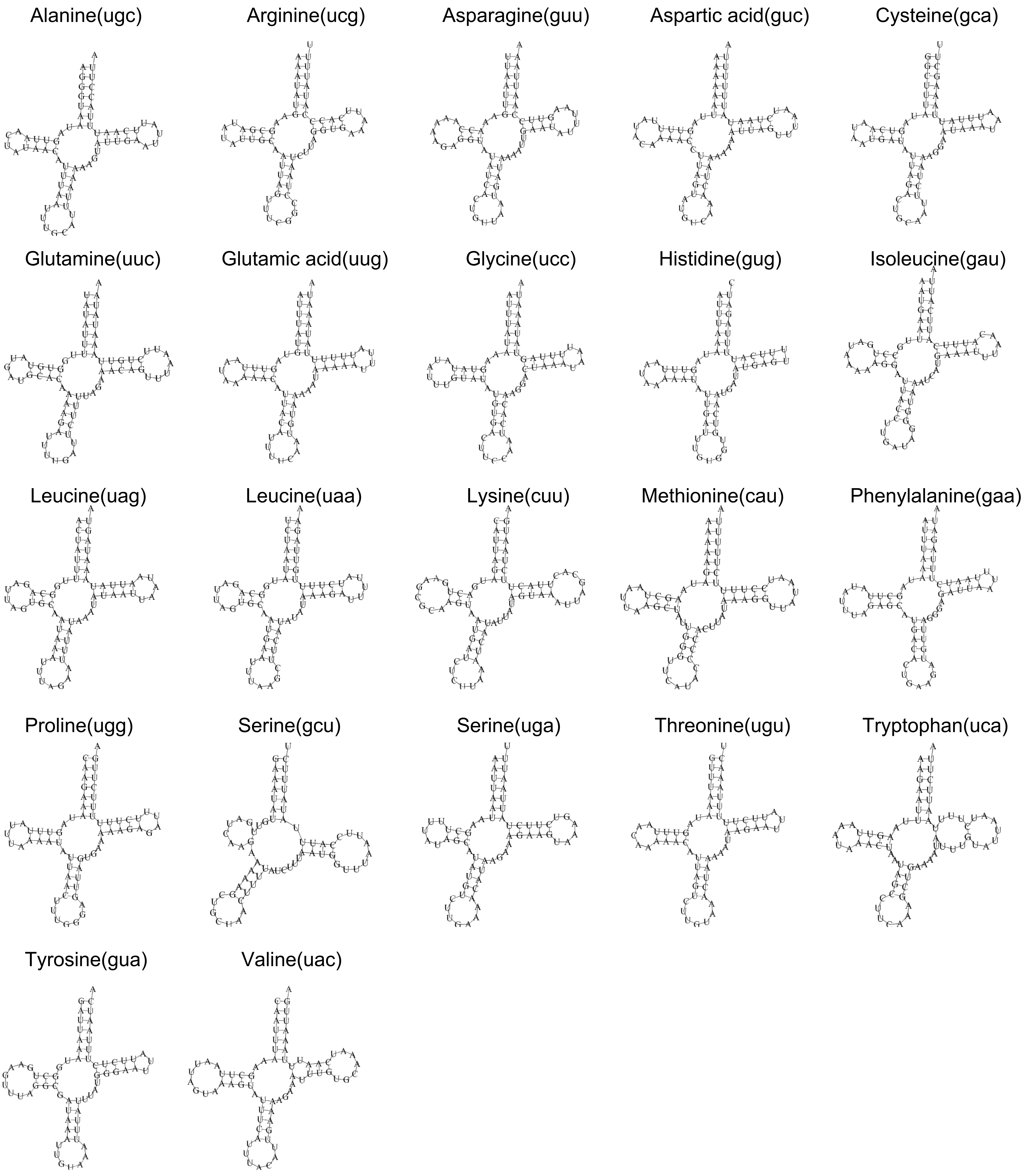

An. kleini

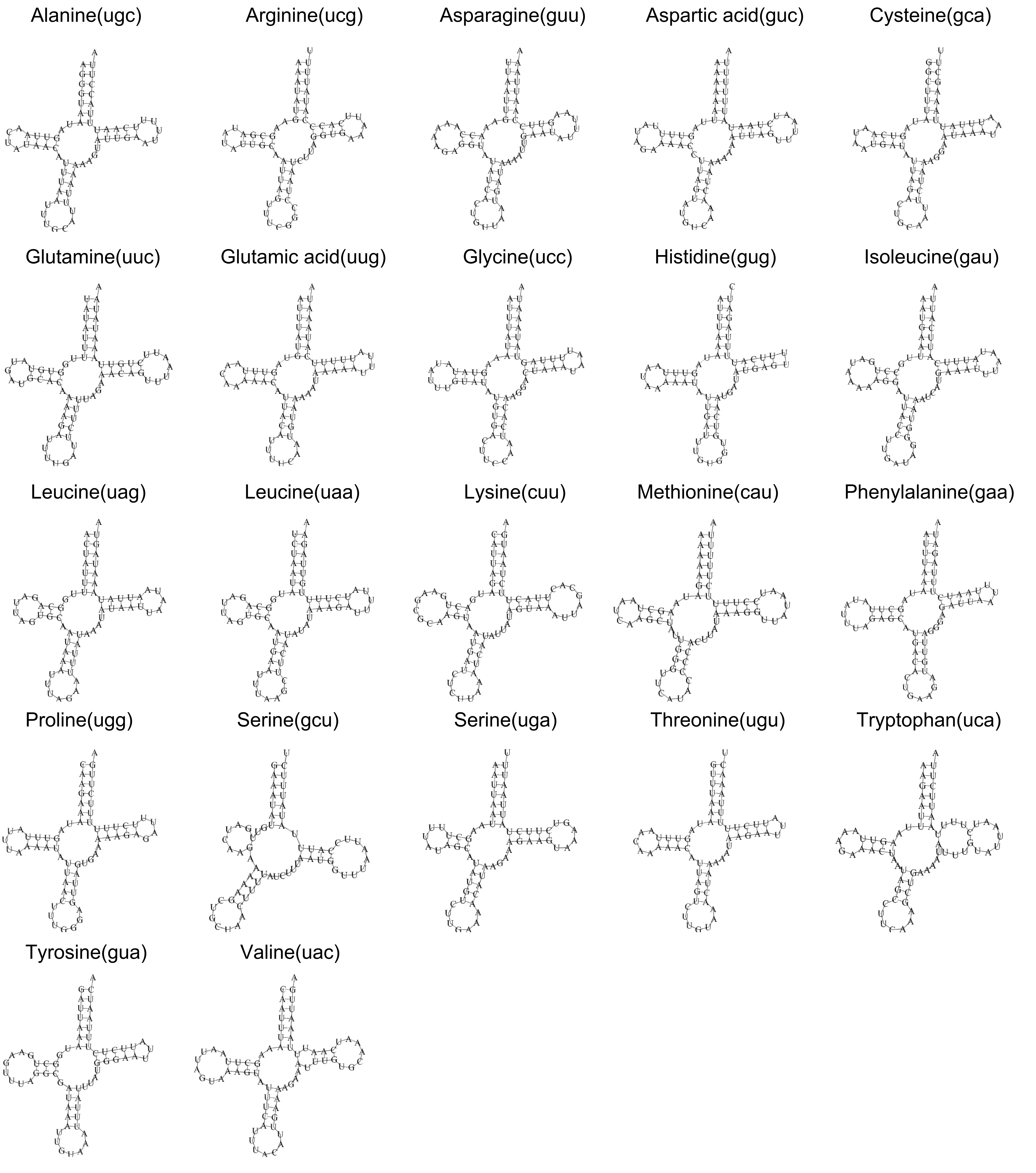
Alanine(ugc)
Arginine(ucg)
Asparagine(guu)
Aspartic acid(guc)
Cysteine(gca)
Glutamine(uuc)
Glutamic acid(uug)
Glycine(ucc)
Histidine(gug)
Isoleucine(gau)
Leucine(uag)
Leucine(uaa)
Lysine(cuu)
Methionine(cau)
Phenylalanine(gaa)
Proline(ugg)
Serine(gcu)
Serine(uga)
Threonine(ugu)
Tryptophan(uca)
Tyrosine(gua)
Valine(uac)

An. kunmingensis

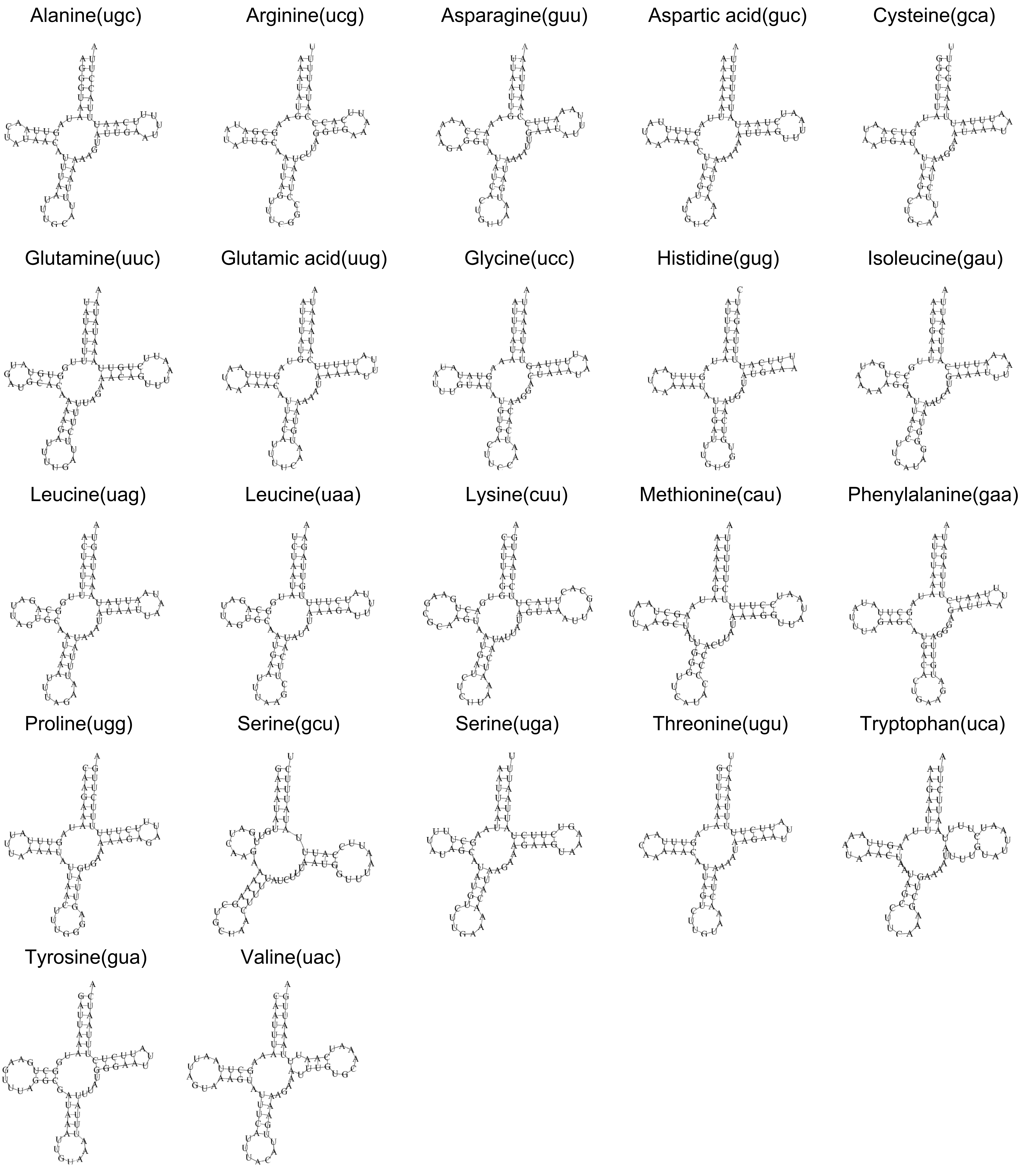

An. lesteri

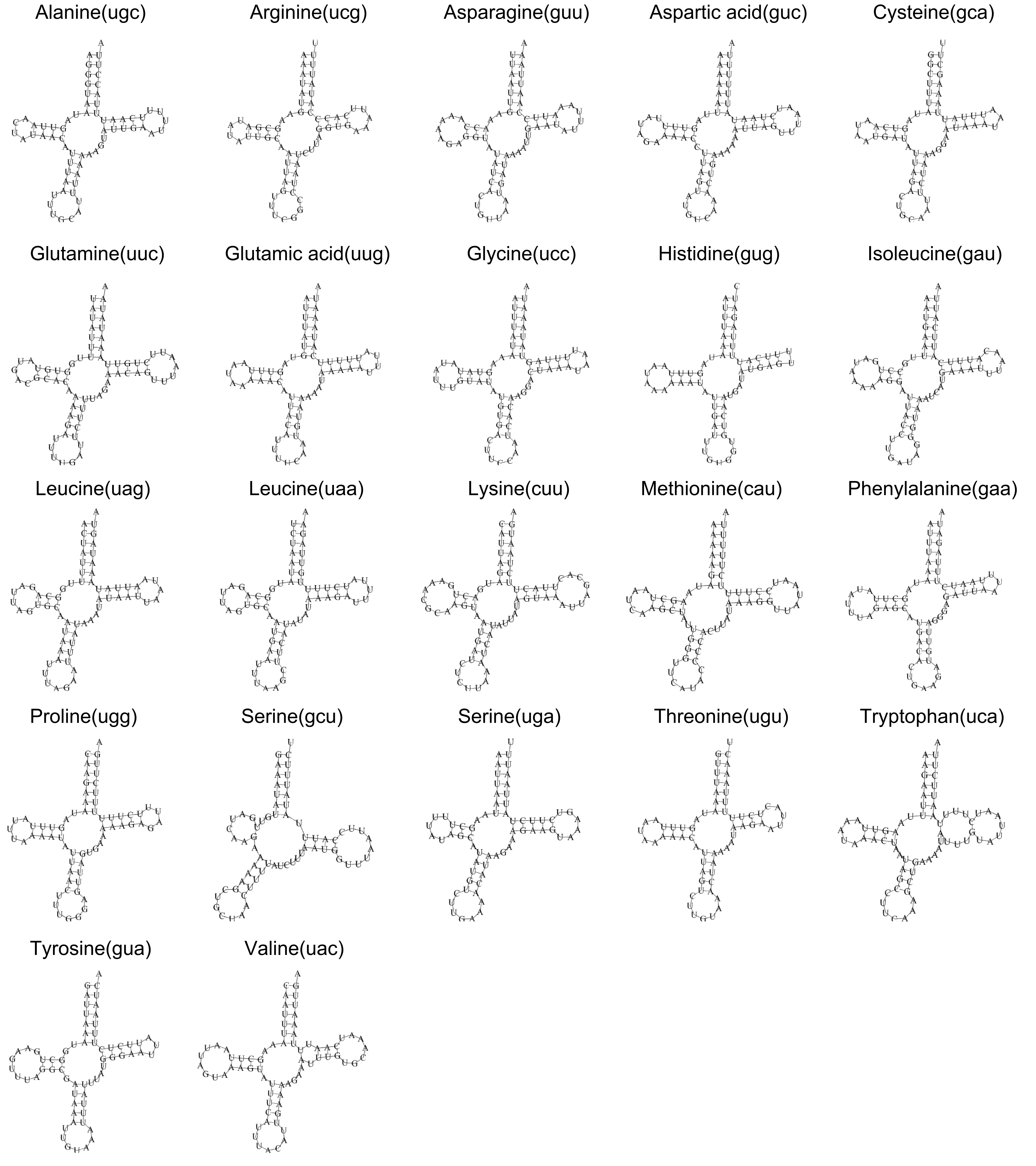

Alanine(ugc)
Arginine(ucg)
Asparagine(guu)
Aspartic acid(guc)
Cysteine(gca)
Glutamine(uuc)
Glutamic acid(uug)
Glycine(ucc)
Histidine(gug)
Isoleucine(gau)
Leucine(uag)
Leucine(uaa)
Lysine(cuu)
Methionine(cau)
Phenylalanine(gaa)
Proline(ugg)
Serine(gcu)
Serine(uga)
Threonine(ugu)
Tryptophan(uca)
Tyrosine(gua)
Valine(uac)

An. liangshanensis

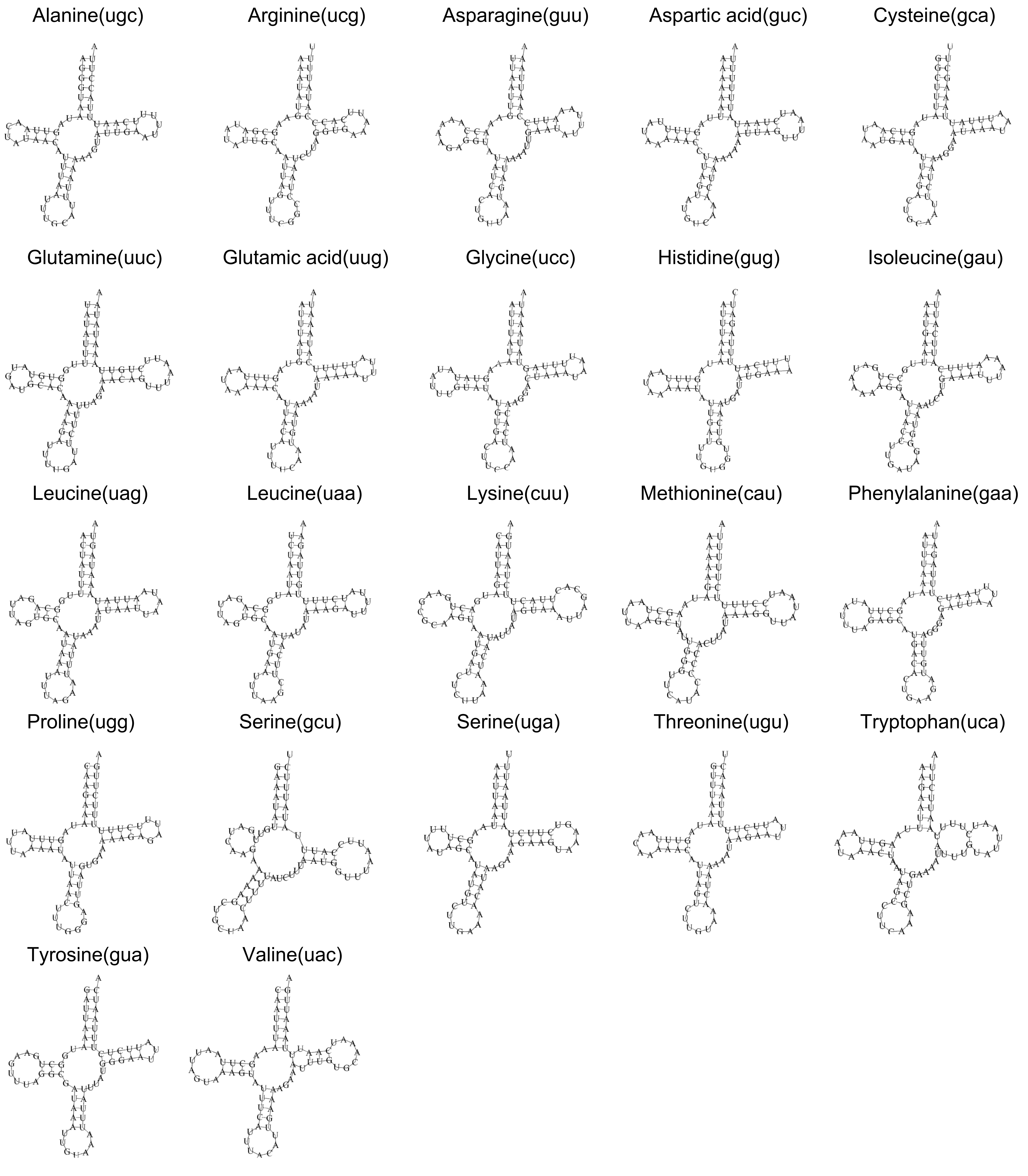

An. peditaeniatus

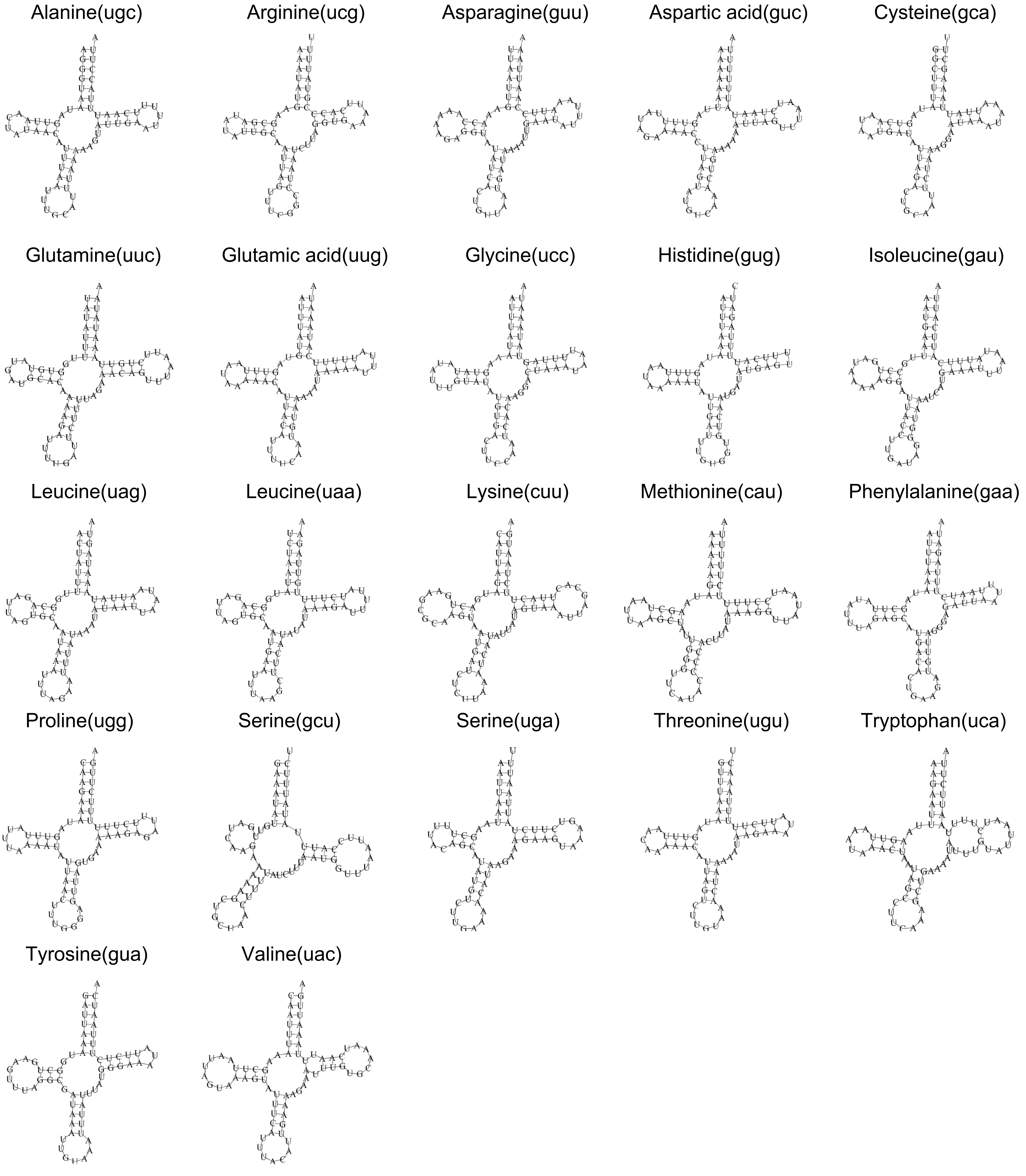

An. sinensis

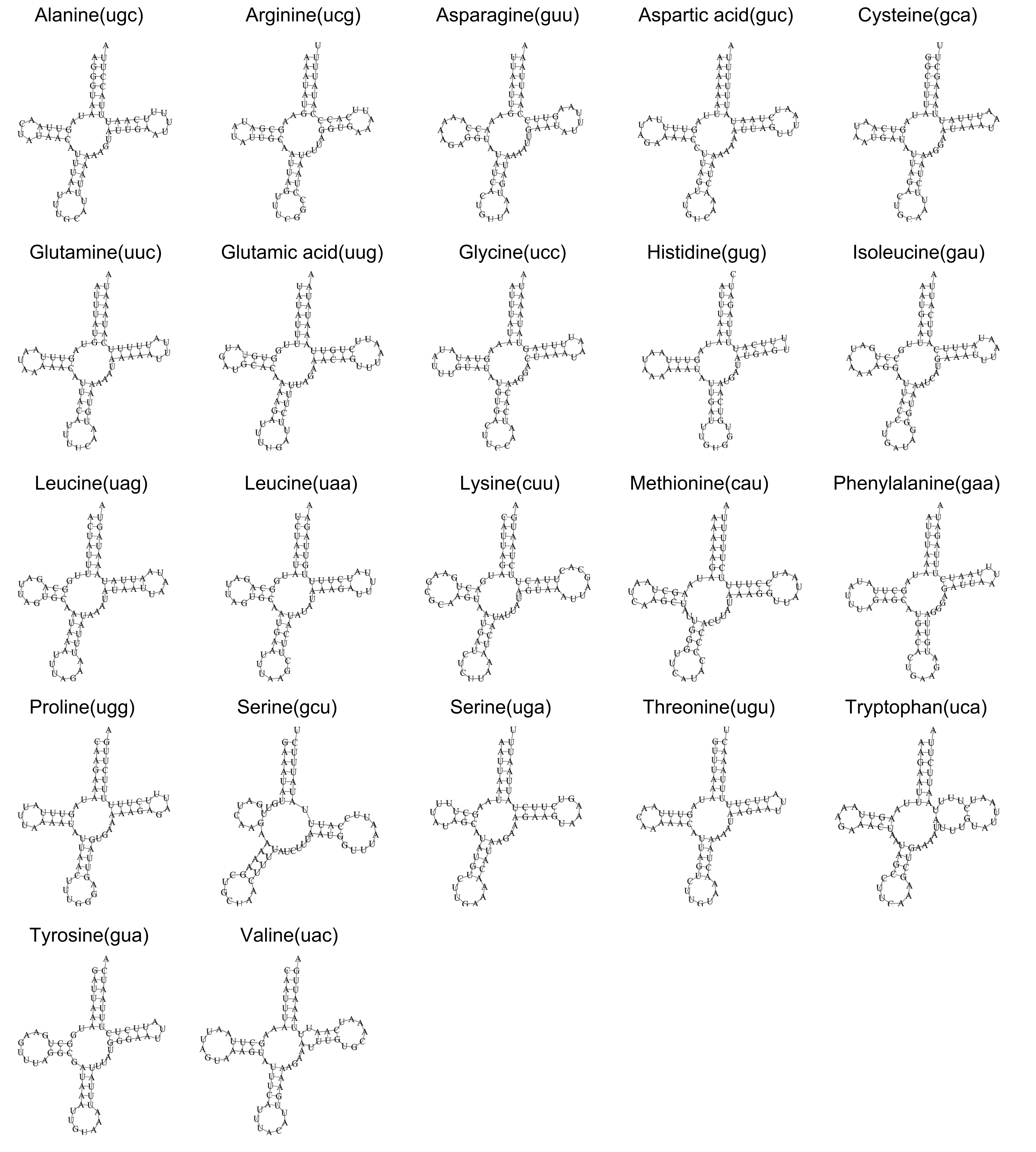

Alanine(ugc)
Arginine(ucg)
Asparagine(guu)
Aspartic acid(guc)
Cysteine(gca)
Glutamine(uuc)
Glutamic acid(uug)
Glycine(ucc)
Histidine(gug)
Isoleucine(gau)
Leucine(uag)
Leucine(uaa)
Lysine(cuu)
Methionine(cau)
Phenylalanine(gaa)
Proline(ugg)
Serine(gcu)
Serine(uga)
Threonine(ugu)
Tryptophan(uca)
Tyrosine(gua)
Valine(uac)

An. yatsushiroensis

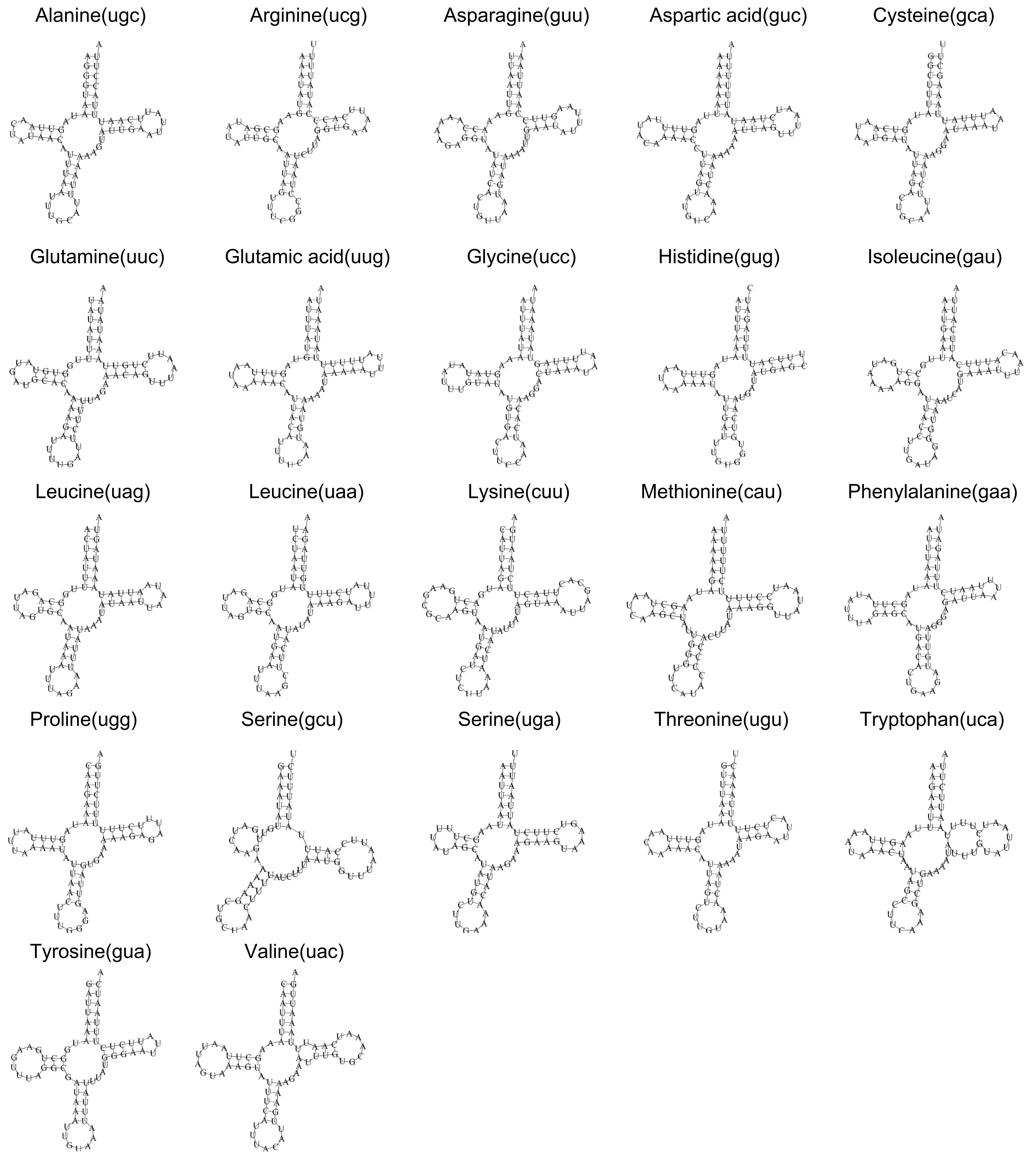

Supplement: Supplementary file 1 [file genes-14-01453-s001.zip › Fig. S3.pdf]
