# Supplementary material for: Phylogenetic Analysis of Some Species of the Anopheles hyrcanus Group (Diptera: Culicidae) in China Based on Complete Mitochondrial Genomes
Source: Genes (Basel). 2023 Jul 16;14(7):1453. doi: 10.3390/genes14071453 (PMC10379722; doi:10.3390/genes14071453)
Supplement: Supplementary file 1 [file genes-14-01453-s001.zip › Fig. S4.pdf]

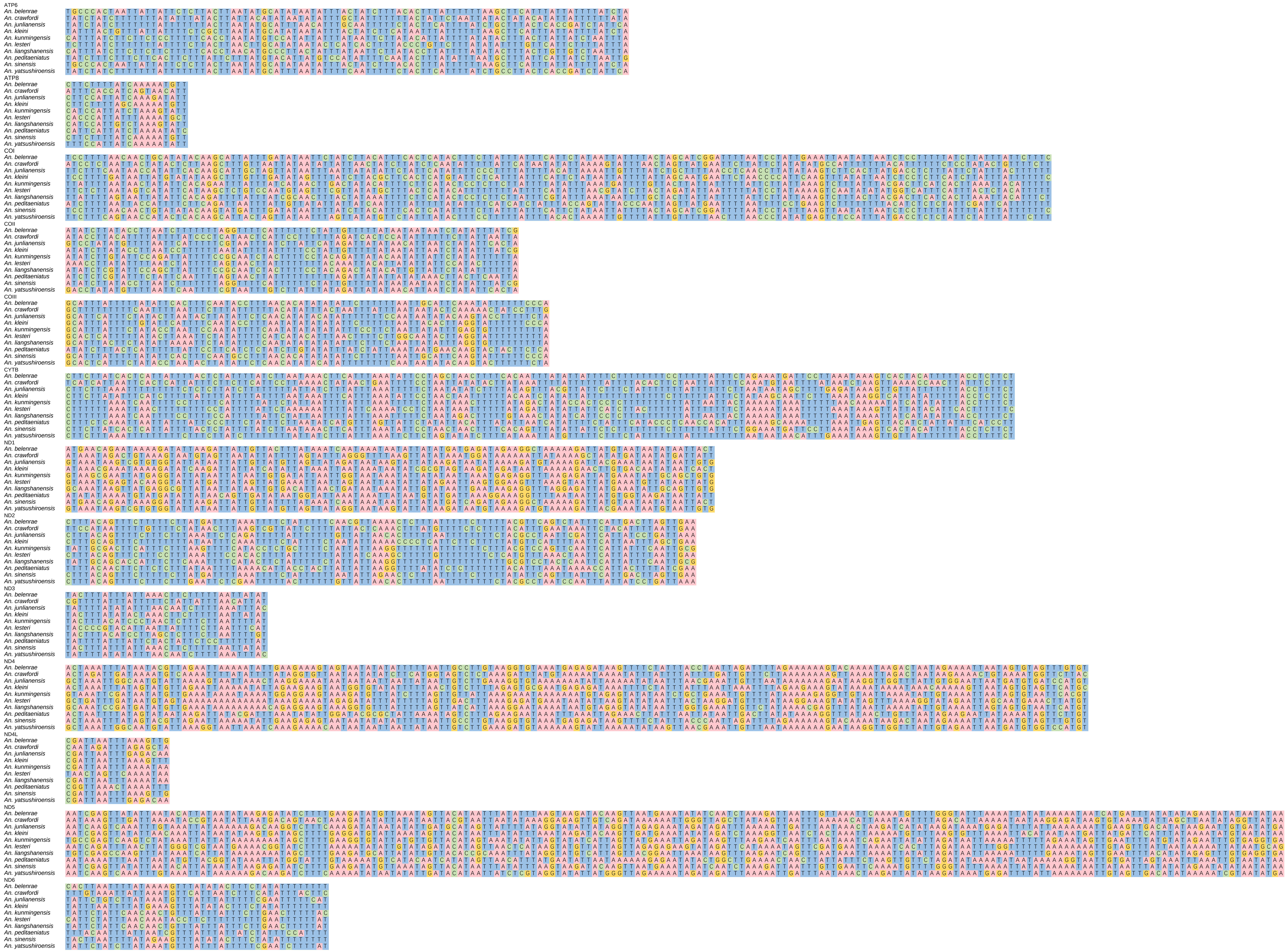
ATP6
ATP8
COI
COII
COIII
CYTB
ND1
ND2
ND3
ND4
ND4L
ND5
ND6
An. belenrae
An. crawfordi
An. junlianensis
An. kleini
An. kunmingensis
An. lesteri
An. liangshanensis
An. peditaeniatus
An. sinensis
An. yatsushiroensis
