# Supplementary material for: Phylogenetic Analysis of Some Species of the Anopheles hyrcanus Group (Diptera: Culicidae) in China Based on Complete Mitochondrial Genomes
Source: Genes (Basel). 2023 Jul 16;14(7):1453. doi: 10.3390/genes14071453 (PMC10379722; doi:10.3390/genes14071453)
Supplement: Supplementary file 1 [file genes-14-01453-s001.zip › Fig. S7.pdf]

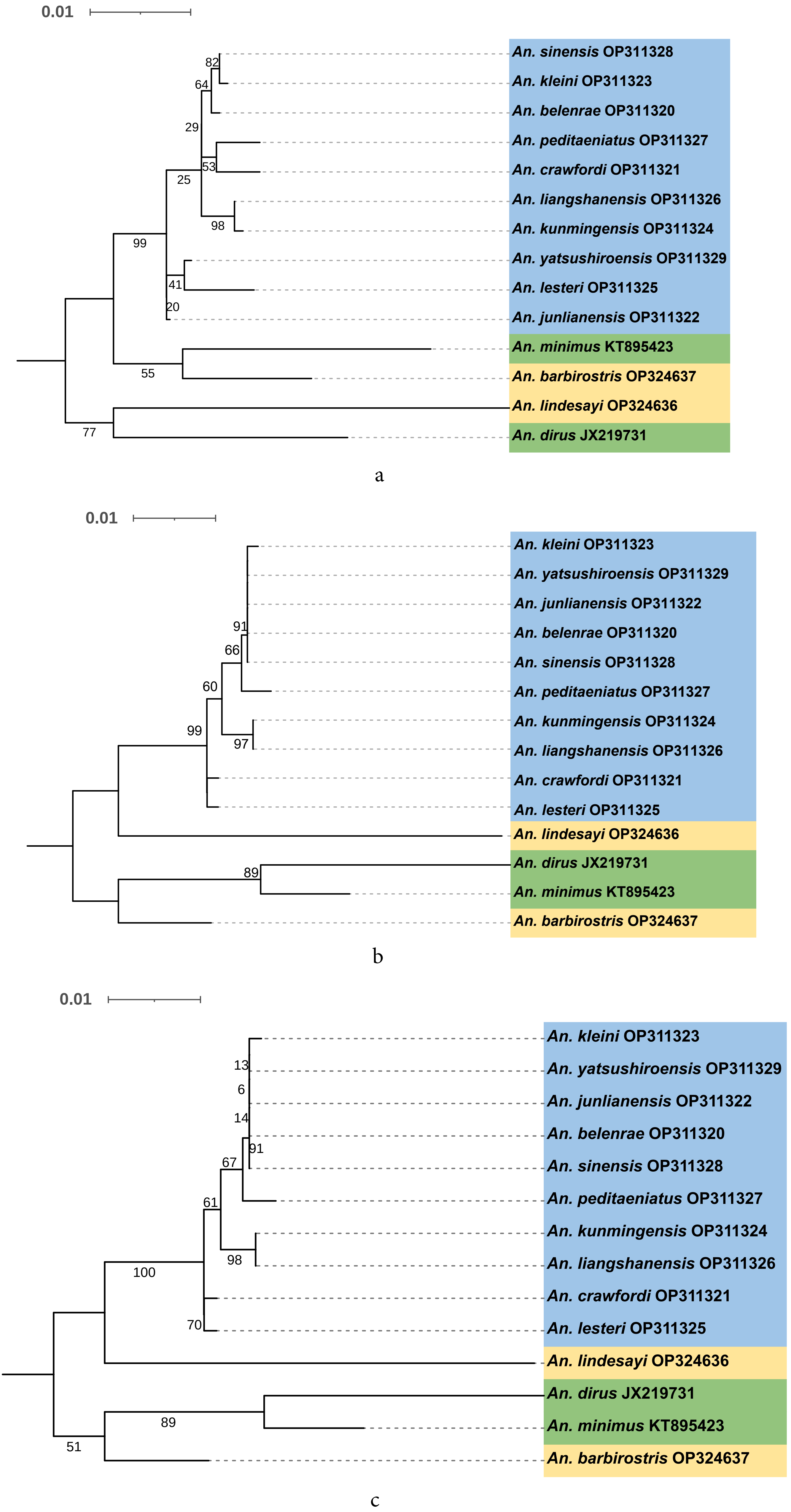

Fig. S6 Phylogenetic reconstruction by maximum likelihood based on tRNA and rRNA coding region concatenated of the species sequenced in this study and two other taxa with data available from GenBank. a tRNAs. b 12S rRNA c 16S rRNA. The values of bootstrapping support are shown to the left of the branch point.
