# Supplementary material for: Phylogenetic Analysis of Some Species of the Anopheles hyrcanus Group (Diptera: Culicidae) in China Based on Complete Mitochondrial Genomes
Source: Genes (Basel). 2023 Jul 16;14(7):1453. doi: 10.3390/genes14071453 (PMC10379722; doi:10.3390/genes14071453)
Supplement: Supplementary file 1 [file genes-14-01453-s001.zip › Fig. S8.pdf]

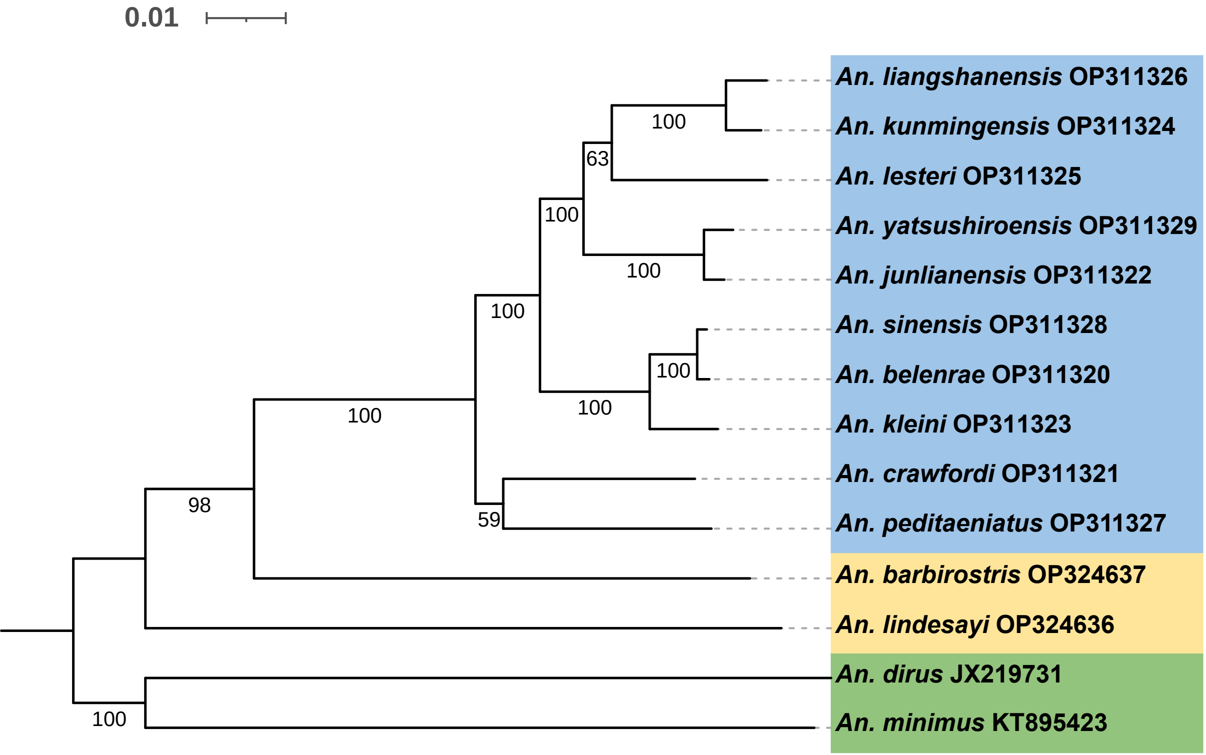

Fig. S7 Phylogenetic reconstruction by maximum likelihood based on the complete mitochondrial genome sequence concatenated of the species sequenced in this study and two other taxa with data available from GenBank. The values of bootstrapping support are shown to the left of the branch point.
