# Supplementary material for: Phylogenetic Analysis of Some Species of the Anopheles hyrcanus Group (Diptera: Culicidae) in China Based on Complete Mitochondrial Genomes
Source: Genes (Basel). 2023 Jul 16;14(7):1453. doi: 10.3390/genes14071453 (PMC10379722; doi:10.3390/genes14071453)
Supplement: Supplementary file 1 [file genes-14-01453-s001.zip › Supplement Tables.pdf]

Table S1. Collection and sequencing information of *Anopheles lesteri* in this study

Species	Collection site	Year	Coordinate	Row data reads	Clean data reads	Mapping reads	Mean depth
An. lesteri(Ⅰ)	Pujiang City, Sichuan Province, China	2006	103.51°E, 30.20°N	57,068,184	56,937,040	266,795	2,553
An. lesteri(Ⅱ)	Pujiang City, Sichuan Province, China	2006	103.51°E, 30.20°N	93,053,680	91,412,210	88,693	700
An. lesteri(Ⅲ)	Pujiang City, Sichuan Province, China	2007	103.51°E, 30.20°N	100,939,412	95,440,544	566,272	4,975

Table S2. Composition of mitochondrial genome obtained in this study

Species	Length(bp)	A%	T%	G%	C%	A+T%	A-T skew	G-C skew
Whole Mitogenome								
An. belenrae	15,418	40.19	38.05	9.20	12.56	78.23	0.027	-0.154
An. crawfordi	15,421	40.33	38.07	9.12	12.48	78.41	0.029	-0.156
An. junlianensis	15,407	40.11	38.04	9.20	12.65	78.15	0.026	-0.158
An. kleini	15,416	40.13	38.13	9.24	12.50	78.26	0.026	-0.150
An. kunmingensis	15,403	40.23	37.95	9.15	12.67	78.17	0.029	-0.161
An. lesteri	15,405	40.27	38.00	9.15	12.58	78.27	0.029	-0.158
An. liangshanensis	15,475	40.12	38.03	9.20	12.65	78.15	0.027	-0.158
An. peditaeniatus	15,413	40.36	37.90	9.17	12.57	78.26	0.031	-0.157
An. sinensis	15,418	40.17	38.10	9.22	12.50	78.28	0.026	-0.151
An. yatsushiroensis	15,406	40.10	38.04	9.22	12.64	78.14	0.026	-0.157
PCGs								
An. belenrae	11,224	33.00	43.82	12.10	11.08	76.82	-0.141	0.044
An. crawfordi	11,224	33.11	43.82	12.07	11.00	76.92	-0.139	0.046
An. junlianensis	11,224	32.93	43.79	12.27	11.01	76.72	-0.142	0.054
An. kleini	11,224	32.96	43.95	12.13	10.97	76.91	-0.143	0.050
An. kunmingensis	11,224	32.89	43.80	12.26	11.05	76.69	-0.142	0.052
An. lesteri	11,224	32.98	43.79	12.23	10.99	76.77	-0.141	0.053
An. liangshanensis	11,224	32.76	43.76	12.33	11.15	76.52	-0.144	0.050
An. peditaeniatus	11,224	32.99	43.86	12.06	11.08	76.85	-0.141	0.042
An. sinensis	11,224	32.96	43.92	12.13	10.99	76.88	-0.143	0.050
An. yatsushiroensis	11,224	32.97	43.78	12.25	11.00	76.75	-0.141	0.054
tRNAs								
An. belenrae	1,476	39.50	38.82	12.47	9.21	78.32	0.009	0.150
An. crawfordi	1,476	39.36	38.82	12.40	9.42	78.18	0.007	0.137
An. junlianensis	1,475	39.46	38.85	12.41	9.29	78.31	0.008	0.144
An. kleini	1,475	39.39	38.71	12.54	9.36	78.10	0.009	0.146
An. kunmingensis	1,474	39.62	38.81	12.35	9.23	78.43	0.010	0.145
An. lesteri	1,476	39.50	38.69	12.40	9.42	78.18	0.010	0.137
An. liangshanensis	1,474	39.69	38.81	12.28	9.23	78.49	0.011	0.142
An. peditaeniatus	1,475	39.53	38.64	12.54	9.29	78.17	0.011	0.149
An. sinensis	1,476	39.43	38.75	12.53	9.28	78.18	0.009	0.149
An. yatsushiroensis	1,475	39.46	38.64	12.41	9.49	78.10	0.010	0.133
rRNAs								
An. belenrae	2,122	38.36	43.03	12.06	6.55	81.39	-0.057	0.296
An. crawfordi	2,123	38.72	43.05	11.73	6.50	81.77	-0.053	0.287
An. junlianensis	2,122	38.22	43.12	12.16	6.50	81.34	-0.060	0.303
An. kleini	2,122	38.36	42.98	12.06	6.60	81.34	-0.057	0.293
An. kunmingensis	2,122	38.45	42.98	12.02	6.55	81.43	-0.056	0.294
An. lesteri	2,123	38.62	43.01	11.82	6.55	81.63	-0.054	0.287
An. liangshanensis	2,123	38.34	43.10	12.01	6.55	81.44	-0.058	0.294

An. peditaeniatus	2,122	37.98	43.26	12.16	6.60	81.24	-0.065	0.296
An. sinensis	2,122	38.36	43.03	12.06	6.55	81.39	-0.057	0.296
An. yatsushiroensis	2,122	38.22	43.07	12.16	6.55	81.29	-0.060	0.300
